# Unmet need for contraception? Understanding postpartum family planning desires and use in Kumasi, Ghana

**DOI:** 10.3389/frph.2025.1625242

**Published:** 2025-09-25

**Authors:** Natalie M. Guzman, Emmanuel Nakua, Cheryl A. Moyer, Jody R. Lori, Veronica Dzomeku, Easmon Otupiri, Sarah D. Compton

**Affiliations:** ^1^ Department of Learning Health Sciences, University of Michigan, Ann Arbor, MI, United States; ^2^ Department of Epidemiology and Biostatistics, School of Public Health, Kwame Nkrumah University of Science and Technology, Kumasi, Ghana; ^3^ Department of Health Behavior and Biological Sciences, University of Michigan School of Nursing, Ann Arbor, MI, United States; ^4^ Department of Nursing, Kwame Nkrumah University of Science and Technology, Kumasi, Ghana; ^5^ Department of Population, Family and Reproductive Health, School of Public Health, Kwame Nkrumah University of Science and Technology, Kumasi, Ghana; ^6^ Department of Obstetrics and Gynecology, University of Michigan, Ann Arbor, MI, United States

**Keywords:** contraception, family planning, postpartum, Ghana, qualitative

## Abstract

**Introduction:**

While most postpartum women in Ghana report they would like to limit or space their births, few are using a highly effective method of family planning. We sought to better understand the reasons behind these seemingly contradictory stances.

**Methods:**

We interviewed 48 postpartum women who had given birth 3–6 months prior and were seeking childhood immunization services at 2 hospitals in urban Kumasi, Ghana. Participants offered their opinions on previous, current, and future family planning use. Interviews were conducted by a trained, bilingual, female research assistant after the infant's appointment in a private room near the Child Welfare Clinic. Interview data were translated and transcribed verbatim and analyzed thematically using NVivo 14.0.

**Results:**

While all participants reported wanting to wait at least 2 years before becoming pregnant again, only 3 were currently using a method of modern contraception. Many of our participants expressed aversion to contraception, driven mainly by the perceived risk of contraception being dangerous to their health and future fertility as reported by members of their social network. Many of those, however, were using either fertility-awareness-based methods, emergency contraception, condoms, or some combination. Those who had had negative personal experience with modern contraceptives were reluctant to use it again due mainly to menstrual side effects. Additionally, some participants had no reason for not wanting to use contraception, they simply do not want to.

**Discussion:**

This qualitative study of women in Kumasi, Ghana, provides a framework to better understand family planning readiness and need. Many participants expressed limited knowledge about modern contraception, highlighting the importance of tailoring counseling to address women's unique questions and concerns. Potential contraceptive users appeared open to and curious about modern methods but had been deterred by stories and misconceptions about adverse consequences. Some women simply chose not to use contraception. Ensuring women have complete, unbiased information on which to base their decisions about contraceptive use and method selection represents a promising avenue for future interventions that seek to improve women's ability to meet their fertility goals.

## 1 Background

While most postpartum women in Ghana are aware of family planning and state that they wish to avoid pregnancy for at least a year (1), 84% who desire to use contraception during this postpartum period do not do so (2). This gap persists despite substantial investment in improving the quality of contraceptive care, including provider training and supply chain enhancements (3–5).

There are likely myriad reasons for the low uptake of modern contraceptive methods among postpartum women. Several factors have been identified by prior research; a woman's own experiences of side effects, health concerns, and side effects reported by members of a woman's social network are routinely stated as reasons for contraceptive non-use in Ghana (6, 7), other low- and middle- income countries (8, 9), and the United States (10). In addition, without proper counseling about side effects and careful matching of methods to individual preferences, women may experience unacceptable adverse effects (5). This phenomenon has been shown to lead to dissatisfaction and discontinuation. While existing quantitative research has identified key barriers to contraceptive use, structured surveys may overlook the nuanced, context-specific perceptions and sociocultural dynamics that shape postpartum women's contraceptive decisions. Qualitative research is uniquely positioned to explore the lived experiences, subjective norms, and community-level factors that influence contraceptive behavior, providing insights that cannot be adequately captured through quantitative methods alone.

In this context, our study aims to deepen our understanding by qualitatively exploring the attitudes and behaviors surrounding family planning among postpartum women in Kumasi, Ghana. We further aimed to learn more about the perceptual gap between the number of women who say they want to limit their number of pregnancies or delay their next birth and the number who are using a method of family planning. By conducting thematic analysis of semi-structured interviews, this research seeks to uncover the complex interplay of beliefs, fears, and social influences that shape decisions around family planning and modern contraceptive use or non-use in this setting. Understanding these factors is crucial for developing health interventions that are both effective and culturally resonant, thereby creating an environment where family planning is accessible and acceptable for postpartum women based on their specific goals and values.

## 2 Materials and methods

### 2.1 Qualitative design

We chose a qualitative approach to enable in-depth exploration of postpartum women's perceptions, desires, decision-making autonomy and interactions with healthcare providers. These elements are central to understanding family planning and contraceptive decision-making in the local context. Additionally, our approach aligns with established principles for using qualitative methods in reproductive health research to capture complex social and cultural influences on health behaviors among women in Ghana (11–13).

### 2.2 Study participants

We recruited postpartum women attending child welfare clinics seeking childhood immunization services at 2 public hospitals representing urban and peri-urban settings in Kumasi, Ghana, to participate in this research. We selected child welfare clinics as recruitment sites because they capture the vast majority of postpartum women. Nearly all mothers bring infants for routine immunizations, regardless of attending their own postpartum visits. Ghana has maintained 90%–95% immunization coverage for the past decade (14). This universal participation makes these clinics ideal for accessing a broad sample of postpartum women. Reproductive-aged women (18–49 years) who had delivered an infant 3–6 months previously and who were comfortable conversing in English or Twi were invited to participate. There were no restrictions to participation based on parity.

### 2.3 Sampling and recruitment

We used convenience sampling to recruit participants. Clinic staff made a general announcement and introduction of the study to all women presenting at the child welfare clinic. Women who met the eligibility criteria (having had a baby within 6 months and able to converse in English or Twi, the local language in Kumasi) and who expressed willingness to participate were referred by clinic staff to the study team for further screening after completing their appointments. Due to this recruitment approach, we are unable to report the number of women who were approached but declined participation, as clinic staff conducted initial screening without documenting refusals. The study team introduced the study to these potential respondents and the consenting women were taken to a private office within the hospital for the interview. Interviewers were bilingual female master's level research assistants trained by the Kumasi-based authors.

### 2.4 Data collection

The interview guide was developed by an experienced international research team consisting of two midwives (one Ghanaian and one American), one reproductive epidemiologist (Ghanaian), and three health services researchers (one Ghanaian, two American). The guide was pilot tested with 5 women at a separate facility, and modifications were made before full implementation. Women were interviewed by trained research assistants using a semi-structured interview guide that included a series of open-ended questions about attitudes and behavior surrounding previous, current, and intended future use of family planning. The interview guide was designed to encourage respondents to express thoughts and feelings that were particularly salient to them. Interviews were conducted in whichever language the participant was most comfortable (English or Twi) by a trained interviewer. Interviews lasted approximately 1 h and were audio recorded, translated to English (if necessary), and transcribed verbatim. While we planned for 50 interviews, we stopped at 48 interviews as data saturation was achieved, and no new themes or insights emerged.

Data collection occurred between September and November 2022. Interviews were conducted evenly across the three-month data collection period. Transcription occurred concurrently with data collection rather than after completion of all interviews, allowing the study team to review and discuss emerging themes to monitor progress toward data saturation.

**Figure 1:**
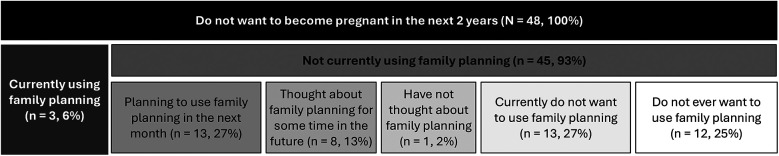
Operationalization of Prochaska and DiClemente's stages of change model for postpartum adoption of family planning in Ghana.

Participants were asked where they believed they fit on Prochaska and DiClemente's Stages of Change model (15). This model reflects a continuum of “readiness” for behavioral change, from those who are not thinking about changing their behavior at all (pre-contemplators) to those who have already changed their behavior and are in the maintenance phase (maintenance). For this study, the interviewer read a series of 6 statements—4 reflecting Stages of Change (15) and 2 the study team felt were important in this context—and asked which most closely fit their current thoughts about family planning. Figure 1 illustrates the six statements used to assess women's stages of change.

Based on the study team's previous experience interviewing women in Ghana, participants were asked about their own experiences but also asked to reflect on hypothetical scenarios written as vignettes. The vignettes were developed based on previous research conducted by the study team and local clinical knowledge from healthcare providers on the team. These vignettes presented narratives drawn from scenarios in the literature and scenarios the research team had previously encountered, including situations where women reported that health workers would not provide contraceptive methods until postpartum bleeding had returned or instances where husbands were unsupportive of contraceptive use.

All study procedures and materials were reviewed and approved by the University of Michigan Institutional Review Board (HUM HUM00263159) and the Kwame Nkrumah University of Science and Technology Committee for Health Research and Publication Ethics. Additionally, administrative approvals were obtained from the health facilities before commencing the study. Each participant was taken through a comprehensive oral consent process before the interviews began. Women received a small token of appreciation for their time (a bar of soap, valued at approximately $1USD).

### 2.5 Data analysis

Transcripts were imported into NVivo 14.0 for thematic analysis following the Attride-Sterling network analysis methodology (16). Transcripts were read and re-read by at least 2 members of the research team. Initial categories for analyzing data were drawn from the interview guide and discussed amongst the research team. A preliminary codebook was developed, which guided the first round of coding. The research team discussed preliminary coding, including the basic themes, organizing themes, and global themes suggested by Attride-Sterling. A second round of coding was undertaken to better reflect higher level themes and patterns that emerged after initial coding.

Following initial coding, the research team discussed preliminary findings, including the basic themes, organizing themes, and global themes suggested by Attride-Sterling. During this process, it became clear that the most distinguishing themes were based on where women placed themselves within the Stages of Change framework, similar to how the 2022 Ghana Demographic and Health Survey reported on contraceptive use and intention (7).

We assigned women to three groups during our iterative analysis process. After the initial coding round, we created data visualizations for each of the six stages of change categories, mapping participants' previous contraceptive experience, methods tried, reasons for hesitancy, spacing goals, and current contraceptive use. These visualizations revealed clear patterns of similarity across specific stages. Participants who had “thought about using family planning,” “planned to start next month,” and “already started using family planning” shared common characteristics and perspectives. Similarly, those who had “not thought at all about family planning” and “did not want to use family planning now” demonstrated comparable attitudes and experiences. The “do not want family planning ever” group exhibited distinct responses that differed markedly from the other categories. Of note, family planning in the context of these statements was synonymous specifically with modern, Western contraceptive methods. Based on these patterns, we consolidated the six stages into three meaningful groups and conducted a second analytical pass (Figure 1). This approach showed that participants within each of the three groups shared qualitatively similar responses, confirming our grouping strategy and enabling more coherent thematic analysis.

A second round of coding was undertaken to better reflect higher level themes and patterns that emerged after initial coding, with additional child codes added to the original parent codes (Supplementary File 1). Both inductive and deductive coding was used in our process. All coding discrepancies were discussed among the research team and consensus was reached.

This manuscript is reported according to the Standards for Reporting Qualitative Research (SRQR).

### 2.6 Reflexivity

Our multidisciplinary team brings together diverse expertise and lived experiences in maternal health, family planning, and reproductive autonomy, with members from both the United States and Ghana. As investigators, we acknowledge that our backgrounds, training, and previous research shape the questions we prioritize, the interpretations we make, and the interventions we seek to implement and evaluate. Our team maintains ongoing dialogue about our positions, privileges, and perspectives, striving for transparency and cultural humility, while remaining committed to centering the voices and preferences of postpartum women in Ghana as the foundation of this work.

## 3 Results

### 3.1 Participant characteristics

A total of 48 postpartum women were interviewed with an average age of 29.5 years and an age range of 19–49 years (Table 1). Additional participant characteristics are included in Table 1.

**Table 1:** Participant characteristics (*N* = 48).

Characteristic	No. (%) or mean ± SD
Age (years)	
Mean ± SD	29.5 ± 6.8
Median	28
Range	19–49
Gravidity	
1	5 (10.4)
2	9 (18.8)
3	14 (29.2)
4	8 (16.7)
5	6 (12.5)
6	2 (4.2)
7	3 (6.3)
8	1 (2.1)
Parity	
1	17 (35.4)
2	8 (16.7)
3	14 (29.2)
4	3 (6.3)
5	4 (8.3)
6	2 (4.2)
Reasons for gravidity-parity mismatch^a^	
Miscarriage	20 (41.7)
Abortion	4 (8.3)
Multiple losses^b^	4 (8.3)
Unknown reason	2 (4.2)
Stillbirth	1 (2.1)
Relationship status	
Married	25 (52.1)
Not married, cohabiting	13 (27.1)
Single	6 (12.5)
In relationship, not cohabiting	1 (2.1)
Married, living separately	1 (2.1)
Unknown	1 (2.1)
Currently using modern contraception^c^	
Yes	3 (6.3)
No	45 (93.8)

^a^ Among participants with gravidity greater than parity (*n* = 31, 64.6%).

^b^ Includes combinations of abortions, miscarriages, and D&C procedures.

^c^ Currently Using Family Planning are women who only responded “I have already started using family planning” to the six statements.

All of the participants said they did not want to be pregnant again within the next 2 years. Very few of the participants were current users of contraception. For women's birth spacing goals, many women wanted to be pregnant in 2–4 years, fewer expressed interest in longer intervals of 5–9 years, and a small number wanted to wait 10 or more years. Some women reported not wanting any more children and a couple women were unsure of their birth spacing goal.

To better understand the perceptual gap in the number of women wanting to delay birth and the number of women using family planning, participants were distributed across the Stages of Change (15): pre-contemplation, contemplation, preparation, and action/maintenance, as well as 2 additional groups added by the study team—ambivalence and refusal.

From those groups, a few participants reported current use of contraception at the time of the interview, many women were planning to start contraception within the next month, some women thought about using contraception sometime in the future, one woman had not thought about it at all, many women did not want to use contraception at the time of the interview, and another large portion of women did not want to ever use contraception.

### 3.2 Group characteristics

**Figure 2:**
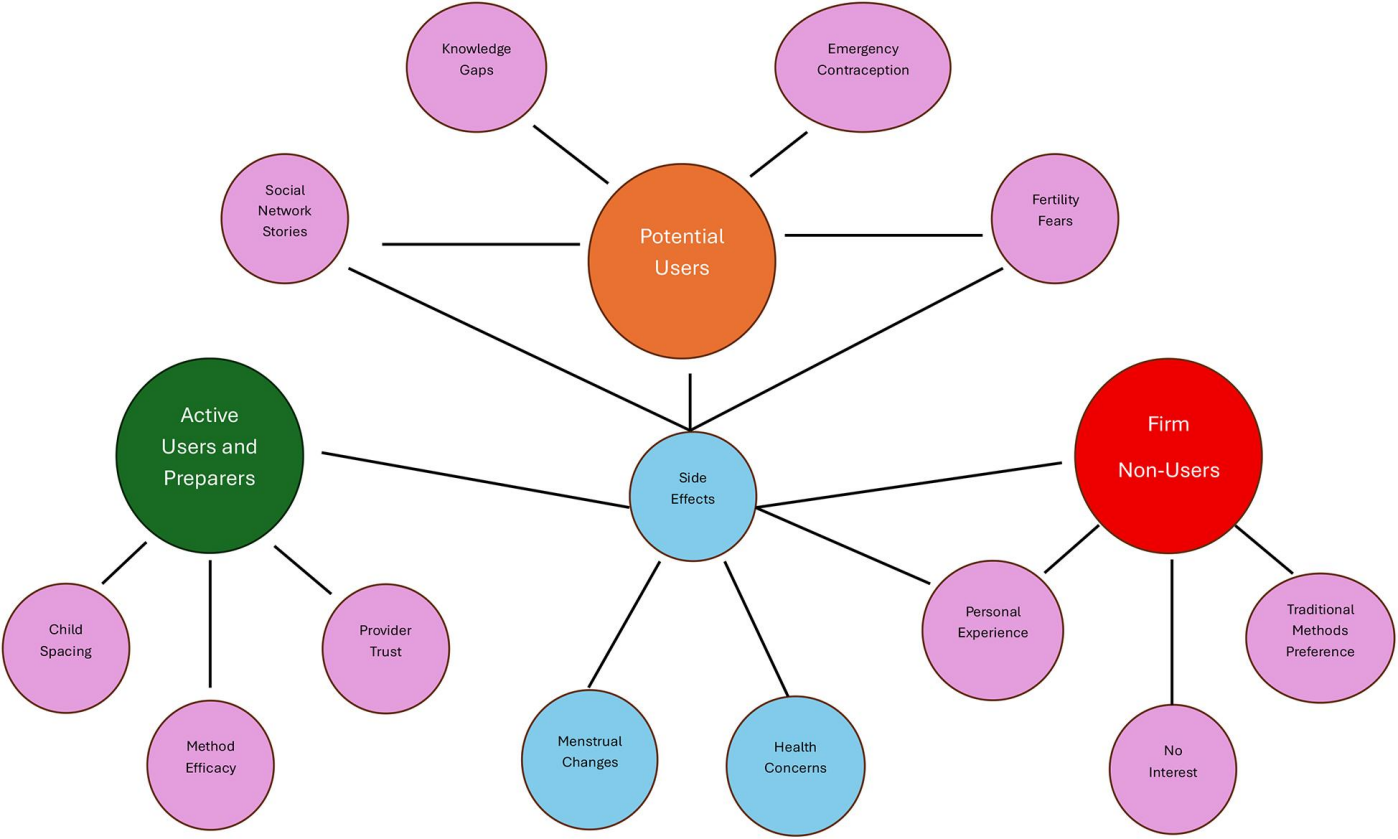
Thematic network visualization showing the interconnected themes across three contraceptive readiness groups among participants. Central blue nodes represent cross-cutting themes affecting all groups, while colored nodes represent group-specific themes: green (Active Users and Preparers), orange (Potential Users), and red (Firm Non-Users). Purple nodes indicate sub-themes within each group.

We found the most meaningful way to understand women's family planning desire was to stratify participants into 3 groups based on our modified Stages of Change model: active contraceptive users and preparers, potential contraceptive users, and firm contraceptive non-users (Figure 1). Figure 2 presents a thematic network visualization of the key themes and their interconnections across the three participant groups.

#### 3.2.1 Active contraceptive users and preparers

These were participants who were already using contraceptive methods, those who intended to start within the next month, and those who were planning to use but not initiating it within the next month. These women appeared at ease with the use of contraception and had well-established routines or plans for using it. For some women, contraceptive methods helped to alleviate their stress related to having an unplanned pregnancy, as they described that having children too close together would cause an extra burden, not allowing them to properly take care of their children:

“[I decided to be on a contraceptive] so that I will not have another baby that fast after this baby so that he will be able to grow well, before I have another baby or else, I will be stressed out.” (Participant 21)

Other women also discussed that with some methods, such as tracking ovulation, puts them at risk for unplanned pregnancy if their husband or partner expresses desire to have intercourse:

“My husband may decide to have intercourse at any time. If you do not use protection, it can happen unplanned.” (Participant 11)

Other women who planned to use contraception also discussed being motivated by the longevity of certain contraceptive methods:

“[People use contraception because] they want to space out their children… and the family planning, you can do the 5-year method, even if you give birth, the other is grown now so you have another baby and you will not face any issues, that is why they do it.” (Participant 42)

While women in this group were now comfortable with contraceptives, one of the participants discussed the importance of a provider taking the time to explain contraception given that she was previously against using it due to perceived side effects:

“I told [the provider] I was extremely scared because I heard when you go in for (contraception) it causes fibroids. So she explained that it was not so… that's what dissipated the fear I had.” (Participant 40)

By finding a provider who was willing to take the time to explain contraceptive methods and discuss misinformation, this woman's perspective on contraceptive methods changed and led her to be comfortable with it. Overall, the women in this group were well-informed about family planning and various contraceptive methods and viewed contraceptives as serving a positive purpose in their life.

#### 3.2.2 Potential contraceptive users

The participants in this group did not want to use contraception now. In contrast to the contraceptive users and preparers, these women were resistant, but not completely opposed to the idea. Their major concern was often related to stories they heard about side effects, but they were unsure if they were true or not. Of the women in the group, the majority self-reported never using family planning, but nearly all the women reported previous use of emergency contraception (EC), condoms, or EC and condoms.

These participants often attributed their reluctance to side effects they heard about from others. One woman described interest in contraception, but reported she had always been deterred due to stories from others:

“I have never used family planning and anytime I decide to, people who have used it before discourage me. Some say you feel dizziness when you walk around and some also look very bad when they use it.” (Participant 13)

An issue that commonly arose was the belief that contraception leads to infertility, which served as a major deterrent for many women:

“The myths around [contraception].. some say even after [contraception], if you want to give birth, it's difficult to get pregnant.” (Participant 35)

“Recently, I went to the family planning unit to undertake [contraception]… She (“the madame”) said for such people [like me], if (providers) do [contraception] for us, it can cut off our fertility completely and you can't give birth again.” (Participant 38)

In addition to fear of infertility, some women were also told that contraception could lead to cancer or death:

“Somebody said when she used [contraception], she got cancer at the genitals.” (Participant 1)

“Oh there was this woman who said she was dizzy, not knowing it was because of the family planning she did. So she didn't get to her destination and then she fainted and died. That's why I don't like it.” (Participant 26)

Overall, in this group, side effects were the main drivers for contraceptive hesitancy. Participants often highlighted the value of enhanced knowledge and education on the side effects of contraceptives, emphasizing that such information would allow women to make informed decisions:

“Some people have some knowledge about it so you talk to them about what it entails and explain to them so they know there is no fear in it.” (Participant 33)

Another participant echoed a similar sentiment when the interviewer asked what could be done to ease the fear related to contraception:

“What can be done is to explain [contraception] to the person for the person to understand very well.” (Participant 27)

Other participants also discussed that women must be proactive if they wish to expand their knowledge of contraception:

“You know this family planning thing, there are rumors people talk about.. but there has not been any intentional announcement or they have not educated women about it unless you walk to a center so they explain to you.” (Participant 1)

“You also have to go to the hospital to get better explanations and use the best option for you.” (Participant 13)

These women both acknowledged that the onus is currently on the woman to learn about contraceptive methods, with Participant 1 explaining that more public announcements could be beneficial to help spread the knowledge. Overall, participants in this group were reluctant to pursue contraception due to stories or advice from others, but they believed that proper explanations and education on family planning and contraceptive methods could make women, including themselves, more comfortable using it.

#### 3.2.3 Firm contraceptive non-users

Participants in this group did not want to ever use contraception and were steadfast in their decision. To meet their fertility goals, these women frequently used the calendar method to track their ovulation cycle (referring to this as “dating”). Among these women, common reasons for not being interested in contraceptive methods included side effects they had personally experienced using family planning previously, perceived side effects, and simply not being interested.

Similar to women who were potentially open to using contraception, side effects were the driving reason for their refusal, but there was a difference in how the participants in each group discussed them. Women who were potential users often brought up “stories” or “myths” of side effects, suggesting they were not completely convinced. Women who were firm non-users stated the side effects much more matter-of-factly, as if they were absolute truths. Such declarative statements included that contraceptives “makes your womb weak” (Participant 17), or that contraceptives “kills you and you will die early when you do it” (Participant 23).

Some women in this group had previous personal experiences that deterred them from contraception. One participant explained that she used the 3-year method and attributed it to causing her fibroid. She described heavy bleeding after removing her implant that alarmed her and led her to go to the hospital:

“Whenever I went to the hospital, they said oh fibroid… so I thought I would never get pregnant again… but I got this pregnancy, so I made up my mind that I will not do [contraception] again.” (Participant 19)

This participant is firm in her stance that contraception caused her to have a fibroid and that she believed she would never get pregnant again. Another woman explained how she had tried multiple types of contraceptives, but that the side effects were not acceptable:

“The reason I don't want to do [contraception]? I’ve done it before, after my first born. I used to do [the] three months type. My menstruation sometimes was on and off and it wasn't flowing well sometimes and what have you. At some point too, I tend to add too much weight so I stopped. So, I was told to use the one month type but when I used it, the same experience occurred. So, I just stopped and it had never occurred to me to use it again and I’ll never use it again.” (Participant 4)

Due to unacceptable side effects for this woman, she refuses to use contraception again.

Another participant echoed a similar sentiment after contracting a yeast infection using contraception:

“I saw that if you use Today (spermicidal sponge), this is the side effect, it gives you that candidiasis and white and so on. So, I stopped using it and I started using the herbal.” (Participant 23)

This woman explains that she tried a contraceptive method, but again, due to unacceptable side effects, decided to use an herbal method instead.

Lastly, there were also many women in this group who had no intention of ever using contraception simply because they were not interested or felt their current method worked well for them:

“I check my days to see the time that I can get pregnant… for me it has never failed me… I am just not interested [in modern contraception].” (Participant 37)

“I do not want to use family planning ever… there is no reason, I just don't want to.” (Participant 41)

“I am not interested in the family planning, I like my date.” (Participant 7)

For these women, their current method satisfies their family planning needs. Overall, women in this group were not open to contraceptive methods because of unacceptable side effects and the point of view that there is simply no reason to switch methods if their current method works for them.

## 4 Discussion

In this qualitative study, we interviewed 48 postpartum women in urban and peri-urban Ghana. While all participants wanted to avoid pregnancy for at least two years, very few were currently using a contraceptive method. Many women had firm plans to begin contraception in the near future, another some did not want to use contraception now but were open to it at some point, and others did not want to use contraception ever.

Our study illuminates important considerations regarding contraceptive terminology and behavior. In these interviews, it became clear that many women who reported never using family planning and stated they did not want family planning had previously used and were currently using EC, condoms, and/or “dating.” According to the World Health Organization (WHO), family planning “allows people to attain their desired number of children and determine the spacing of pregnancies” (17). However, among the women we interviewed, the term “family planning” was used synonymously with modern, preventative contraception, often referring specifically to oral contraception, intrauterine devices (IUDs), and implants. Many women who reported not using family planning were actively pursuing methods to accomplish their desired number of children and birth timing through emergency contraception, physical barriers like condoms, and the calendar rhythm method.

This terminology gap could have major implications for how large-scale surveys are interpreted. As Staveteig (2017) found in qualitative follow-up to the 2014 Ghana Demographic and Health Survey, a large proportion of women who were recorded as having an unmet need for contraception were in fact engaging in contraceptive behavior, mainly traditional methods (18). However, in the decade since that study, there appears to be increased use of EC (7), and women in our study currently using EC, who were mainly in the Potential Users group, did not consider EC to be family planning. Participants in both the Active Users and Potential Users groups considered Western forms of contraception like birth control pills, injections, and intrauterine devices to be family planning. This highlights the importance of qualitative work in this space, as these nuances would be challenging to uncover with quantitative methods alone.

Beyond the under-reporting of contraceptive behavior identified by Staveteig (2017), the concept of “need” within the unmet need construct presents additional challenges (18). Unmet need for family planning, as defined by DHS methodology and standardized by Bradley et al. (2012), refers to the percentage of women who are fertile and sexually active but are not using any method of contraception and report wanting to space their next birth by at least two years or wanting no more children (19). This measure has been critiqued for its complexity and potential to misrepresent women's actual contraceptive needs, as it assumes that all women classified as having “unmet need” would use contraception if it were available. While we are not the first to highlight this issue (20), some women in our study would be classified as having an unmet need according to standard definitions. However, because these women actively choose not to use contraception, they do not, by definition, have a genuine need for it.

A large proportion of our participants expressed a strong aversion to modern contraception. As Staveteig (2017) also found, many of our participants reported the risk of menstrual side effects and personal opposition to contraception due to personal and/or close social contacts' experience of negative side effects as driving reasons for their disinterest (18). While some of this opposition could be averted by comprehensive education on side effects of different family planning methods, including eliciting women's feelings about certain side effects and better matching them with a method that is not likely to produce them (21–23), satisfied non-use must be accepted as a positive outcome of any family planning program (24).

Our findings from Ghana align with broader patterns documented across low and middle-income countries where similar tensions between fertility intentions and contraceptive use persist. A scoping review of quantitative and qualitative studies across low and middle-income countries found that women's fear of side effects or other health concerns related to contraceptive methods was among frequently reported reasons for non-contraception use (25), which mirrors our participants' concerns about menstrual side effects and fertility fears. Interestingly, while machine learning analysis in Ethiopia revealed that “husband/partner disapproval to use family planning” was among the top predictors of unmet need (26), this did not emerge as a prominent theme in our conversations with women in Ghana, though our qualitative work is not meant to be generalizable.

In India, almost 51% of women were not using contraception to delay their next birth despite expressing spacing desires (27), paralleling our observation that all participants wanted to wait at least two years before becoming pregnant again, yet only three were using modern contraception. Similarly in this study, women's autonomy was found to be an important factor in reducing unmet need, consistent with our findings. These findings were also consistent across sub-Saharan Africa where the overall decision-making capacity of women was found to be the sole significant predictor of unmet needs (28). Additionally, another study of unmet need among women in sub-Saharan African countries found that knowledge about modern contraceptive methods was among the individual-level factors that were associated with both the unmet need for spacing and limiting, reinforcing our finding that many participants expressed lack of confidence in their contraceptive knowledge (29).

If the goal of family planning programs is to improve the ability of women to reach their fertility goals, our study can offer important insights. Many of our participants expressed their own lack of knowledge about contraception. While Ghana reports “near universal” knowledge about contraception (7), beyond naming methods, it is clear many women do not feel confident in their knowledge. Postpartum contraceptive counseling is usually done in groups and, while effective at reaching a larger number of women, the quality of this counseling has been questioned (30). Our study shows the importance of tailoring counseling to women's unique questions and concerns. Those in the Potential Users group appear open to contraception, and curious about it, but have heard many stories and “myths” about consequences of contraception. As Agula et al. (2022) found among low-income women in Accra, Ghana, there may be opportunities to enhance the quality of counseling provided during antenatal and postnatal care visits, thereby ensuring that clients are able to make fully informed decisions regarding postpartum contraception (31).

**Figure 3:**
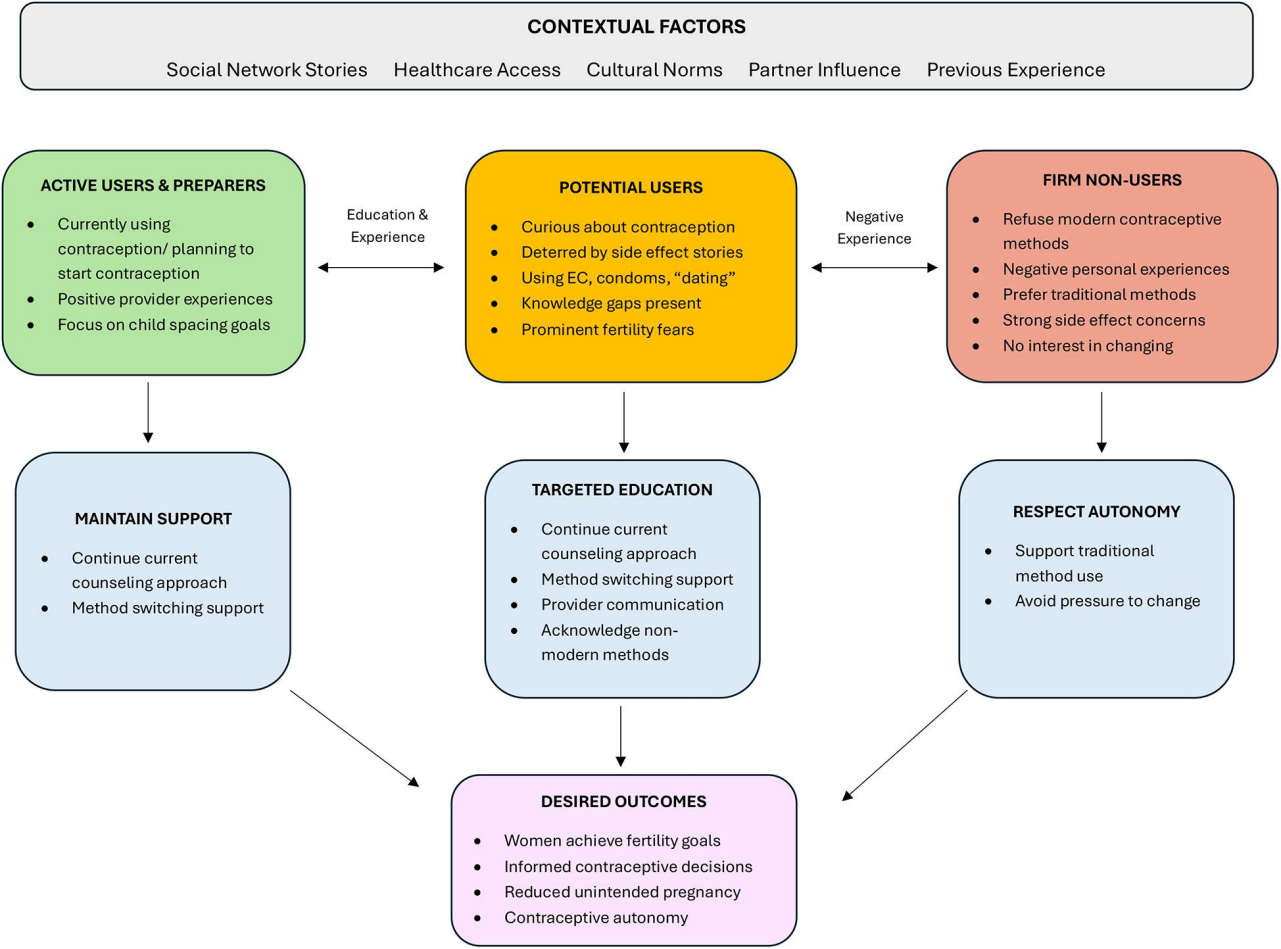
Conceptual framework for postpartum contraceptive decision-making. Conceptual framework illustrating the decision-making pathways for postpartum contraceptive use among participants. This framework demonstrates how contextual factors influence placement within three distinct readiness groups, each requiring tailored intervention approaches to achieve desired outcomes. Dashed bidirectional arrows indicate potential movement between groups based on education, experiences, and changing circumstances. The framework emphasizes that different groups require different counseling approaches, with Potential Users representing the greatest opportunity for evidence-based education interventions, while respecting the autonomous choices of Firm Non-Users who prefer traditional methods.

The objective of improved counseling and education should not be to persuade women to initiate family planning use, but rather to ensure they are making well-informed decisions. We have provided a conceptual framework for understanding contraceptive decision-making pathways that can serve as a foundation for developing targeted interventions that optimize support for women across different stages of contraceptive readiness (Figure 3). Overall, our findings suggest family planning counseling should center women's preferences and provide comprehensive education about all contraceptive options, including non-hormonal methods for women and their partners, to better enable women to achieve their fertility goals. This approach supports women's healthcare decision-making, regardless of whether they choose to utilize Western methods of family planning.

Our findings call into question the utility of current measures used in surveys to assess usage. We found ambiguity in language around family planning—some of our participants reported not using family planning and not wanting to use family planning, yet reported using EC, which is a hormonal form of contraception. This was revealed only after asking explicitly what they were doing to prevent pregnancy after many reported not using any method of family planning. Our results suggest it may be more useful to ask, “What are you and/or your partner doing to avoid pregnancy?” rather than simply whether women are using contraception. Additionally, qualitative follow-up studies, as the DHS has conducted in Ghana, could assess whether contraceptive non-use is undercounted (18).

### 4.1 Limitations

Our study should be interpreted in the context of several limitations. Ours was not a random sample and was not designed to be representative. Although we believe selection bias is minimal given that most women seek health services for their infants, we lack information about participants who declined to join the study. Consequently, it is possible that women who chose to participate differ substantially from those who declined. The 48 women were recruited from 2 hospitals in one part of Ghana. While we believe we have accurately presented what they reported to us, we do not suggest these thoughts and feelings represent all postpartum women in Ghana.

Our study also has many strengths. The long interviews meant that women were able to express their thoughts and feelings thoroughly. The use of vignettes has been shown to be an effective way to encourage Ghanaian women to speak more— reflecting not only on the characters in the story, but also on how those relate to their own lives. Our study also expanded upon previous conceptualizations of family planning use/non-use. By incorporating the Stages of Change Model to identify where women are in terms of behavior change, we expanded the approach to unpacking women's attitudes and provided a more nuanced understanding than has been seen previously in this context.

### 4.2 Conclusion

In this qualitative study of postpartum women in Kumasi, Ghana, we found that women's attitudes toward modern contraceptive family planning were shaped by three distinct stages of readiness: active users and preparers who valued contraception for spacing goals, potential users who were curious but deterred by stories of side effects, and firm non-users who preferred traditional methods such as dating or had experienced unacceptable side effects. While all participants wanted to delay pregnancy for at least two years, few were using modern contraceptive methods, with many relying on emergency contraception, condoms, or fertility awareness methods they did not consider “family planning.” These findings suggest that efforts to improve postpartum contraceptive counseling should consider women's readiness for change and provide accurate information to address misconceptions, while respecting women's autonomous choices about contraceptive use.

## Data Availability

The raw data supporting the conclusions of this article will be made available by the authors, without undue reservation.
